# Effect of Fe on the Hydrogen Production Properties of Al-Bi-Sn Composite Powders

**DOI:** 10.3390/ma15196702

**Published:** 2022-09-27

**Authors:** Cuiping Wang, Bohao Yin, Kairui Lin, Mingshuai Wang, Rui Deng, Yihui Guo, Jinbin Zhang, Shuiyuan Yang, Xingjun Liu

**Affiliations:** 1 Fujian Key Laboratory of Surface and Interface Engineering for High Performance Materials, College of Materials, Xiamen University, Xiamen 361005, China; 2 Xiamen Key Laboratory of High Performance Metals and Materials, Xiamen University, Xiamen 361005, China; 3 School of Materials Science and Engineering, Institute of Materials Genome and Big Data, Harbin Institute of Technology, Shenzhen 518055, China; 4 Shenzhen R&D Center for Al-Based Hydrogen Hydrolysis Materials, Shenzhen 518055, China

**Keywords:** hydrolysis reaction, hydrogen generation, gas atomization, Al-based powder

## Abstract

Fe additives may play an important role in the preparation of aluminum-based hydrolysis hydrogen powder, with high hydrogen yield, low cost, and good oxidation resistance. Therefore, it is necessary to ascertain the effect of Fe on the hydrogen production performance of Al-Bi-Sn composite powders. According to the calculated vertical cross-section of the Al-10Bi-7Sn-(0~6)Fe (wt.%) quasi-binary system, Al-10Bi-7Sn-xFe (x = 0, 0.5, 1.5, 3) wt.% composite powders for hydrogen production were prepared by the gas-atomization method. The results showed that the Al-10Bi-7Sn-1.5Fe (wt.%) powder exhibited an extremely fast hydrogen generation rate at 50 °C, which reached 1105 mL·g^−1^ in 27 min in distilled water, 1086 mL·g^−1^ in 15 min in 0.1 mol·L^−1^ NaCl solution, and 1086 mL·g^−1^ in 15 min in 0.1 mol·L^−1^ CaCl_2_ solution. In addition, the antioxidant properties of these powders were also investigated. The results showed that the hydrogen production performance of the Al-10Bi-7Sn-1.5Fe (wt.%) powder could retain 91% of its hydrogen production activity, even though the powder was exposed to 25 °C and 60 RH% for 72 h. The addition of Fe not only promoted the hydrogen generation rate of the Al-Bi-Sn composite powders, but also improved their oxidation resistance. The Al-10Bi-7Sn-1.5Fe (wt.%) composite powder shows great potential for mobile hydrogen source scenarios with rapid hydrogen production.

## 1. Introduction

Energy shortages and environmental pollution caused by fossil fuels have been enormous challenges for the development of energy societies [1,2,3,4]. Seeking a sustainable and alternative energy source to replace traditional fossil fuels is the priority at present. Hydrogen energy has been widely regarded as one of the most promising alternative energy sources due to its high-combustion calorific value, nonpolluted reaction products, and abundant natural sources [5,6,7,8]. Many methods have been used to produce hydrogen [8,9,10,11,12,13]. The metal hydrolysis reaction provides an immediate and effective method to generate hydrogen [9,10,11,12,13], which can instantly generate hydrogen with water, thus avoiding the storage and transportation of hydrogen from the root [10,14,15]. Aluminum is an ideal hydrogen production source that possesses excellent properties such as lower price, abundant resources, and high theoretical hydrogen release rates [16,17,18]. Theoretically, 1 g of Al yields 1.36 L of H_2,_ assuming 100% hydrolysis at standard ambient conditions. Further, the reaction of Al hydrolysis can be expressed as follows [19]:

2Al + 6H_2_O → 2Al (OH)_3_ + 3H_2_ (1)

2Al + 4H_2_O → 2AlO (OH) + 3H_2_ (2)

Nevertheless, Al exposed to air forms a dense oxide film, which impedes the inner Al and water [20,21]. Many studies have tried to find suitable physical or chemical methods to destroy the oxide layer on the aluminum surface [8,16,21]. Many works have proved high-energy ball milling to be an effective way for activating Al, which significantly increases the specific surface area and the defects on the powder surface [22,23,24,25,26,27,28,29]. However, the surface protection of the ball milling Al-based composite powders is essential to prevent the formation of an Al_2_O_3_ passivation layer. Recently, it has been reported that alloying and gas atomization methods are good strategies to optimize the hydrogen generation properties of Al-based alloys [30,31,32,33,34]. A small amount of alloying elements can improve hydrogen production characteristics by modifying Al-based alloys’ microstructure and phase compositions [33,34,35]. In practical applications, Al-based composite powders are usually alloyed with metals, such as Bi, In, Ga, and Sn, to break the oxide layer and thus improve their hydrogen generation properties [22,25,28,29]. The low-melting point metals Bi and Sn easily form numerous holes, gaps, and other defects on the surface of Al, which increases the contact area between Al and water during hydrogen production by hydrolysis, thus improving the hydrogen production performance of the alloy [31,32,33,34,35,36,37,38,39]. Previous studies showed that core-type macroscopic morphology is formed by the addition of Bi and Sn in the Al-base alloys [40]. The alloy composite powders prepared by the gas atomization method have excellent hydrogen production performance by hydrolysis and can generate a large amount of hydrogen in a short time [31]. Liu et al. [23] prepared Al-Bi and Al-Sn binary alloy composite powder by the gas atomization method, and Wang et al. [26] prepared Al-Bi-Sn ternary alloy composite powder in same way.

However, when the alloy composite powder is placed in the air for a certain time, it will lead to oxidation inactivation, which will significantly reduce its hydrogen production performance by hydrolysis [22,25]. At the same time, the low-melting point metals Bi and Sn are expensive, which increases the cost of hydrogen production [31]. Researchers have tried to solve the above problems. Recently, it has been reported that a homogeneous macroscopic morphology is formed by the addition of Fe [40], which may help to improve the hydrogen production performance of Al hydrolysis. In addition, the replacement of primary aluminum with waste aluminum containing small amounts of Fe, Cu, and Sn has resulted in an even further price reduction of 30% [41]. Unfortunately, no studies have yet reported the use of Fe as an additive to reduce the cost of materials for gas-atomization hydrogen-production powders.

In this study, Bi, Sn, and Fe were selected as additives to the aluminum alloy in the present research to optimize the hydrogen production performance and antioxidant properties of Al-based composite powder and control material cost. The Al-10Bi-7Sn-0.5Fe, Al-10Bi-7Sn-1.5Fe, Al-10Bi-7Sn-3Fe, and Al-10Bi-7Sn (wt.%) composite powders were designed by calculated phase diagram and prepared by gas atomization pulverization technology. The morphologies and structures of the prepared powders were characterized and analyzed. Meanwhile, the hydrogen generation performances of these prepared gas-atomization metal powders in different aqueous solutions, such as distilled water, NaCl solution, and CaCl_2_ solution, were investigated. Furthermore, the hydrogen generation properties after exposure to air were also investigated.

## 2. Experimental Procedures

### 2.1. Powder Preparation

In this study, the gas atomization method was used to prepare Al-10Bi-7Sn-xFe (x = 0, 0.5, 1.5, 3) wt.% composite powders. The specific preparation processes of the composite powders were as follows: Al (purity: 99.9%), Bi (purity: 99.9%), Sn (purity: 99.9%), and Fe (purity: 99.9%) bulks were inductively pre-melted by a high-frequency induction melting furnace in an alumina crucible under argon gas protection, and then atomized by argon gas flow at a high pressure (8 MPa). The molten metal alloy material temperature should be 80 °C higher than the liquid miscibility gap to obtain a uniform liquid phase. After atomization, the abnormally coarse particles were filtered out by sieves in the argon gas environment, and then the powders were respectively loaded into labeled glass vials for subsequent experiments.

### 2.2. Hydrogen Generation Measurement

**Figure 1:**
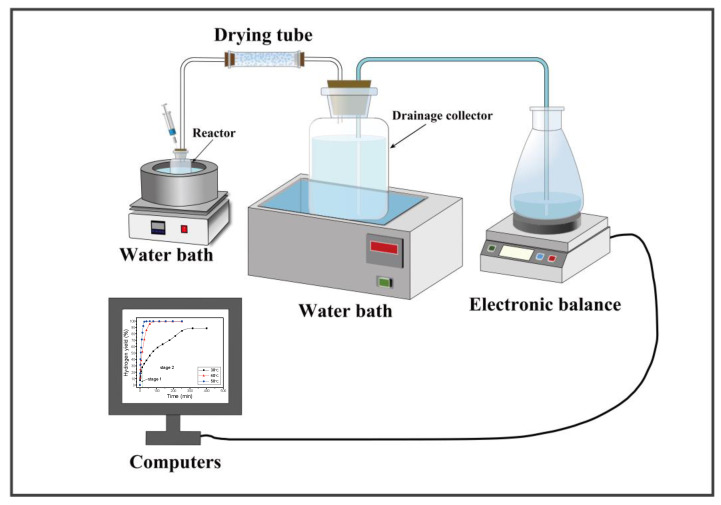
The experimental system used for measuring hydrogen production.

The hydrogen production performance of the composite powders was determined by the drainage method [23]. A schematic diagram of the hydrogen production measurement device is shown in Figure 1. A 125 mL flask reactor containing 0.3 g alloy composite powder was placed in a water bath and continuously stirred. After the temperature (30 °C, 40 °C, 50 °C) was stable, 10 mL preheated reaction medium, such as distilled water, was injected into the reactor (preheated until the temperature was stably consistent with the reaction temperature). The hydrogen produced by alloy composite powder hydrolysis was purified through a drying tube to remove moisture. The actual hydrogen production was obtained indirectly by measuring the weight of the replaced water, and the hydrogen conversion efficiency of the material was the ratio of the actual hydrogen production to the theoretical value.

### 2.3. Characterization Methods

In this study, a scanning electron microscope (SEM, SU70, Hitachi, Tokyo, Japan) was used to observe the microscopic morphology of the composite powders. An energy-dispersive X-ray spectrometer connected to the SEM was also used to determine the element distribution and composition. Particularly, the powers were measured after being mounted, ground, and polished, when observing the microstructure of the internal cross-section of the composite powder. An X-ray diffractometer (XRD, D8, Advance, Bruker, Madison, WI, USA) was used in this study to analyze the crystal structure of the composite powders.

## 3. Results and Discussion

### 3.1. Design of Powder Composition

**Figure 2:**
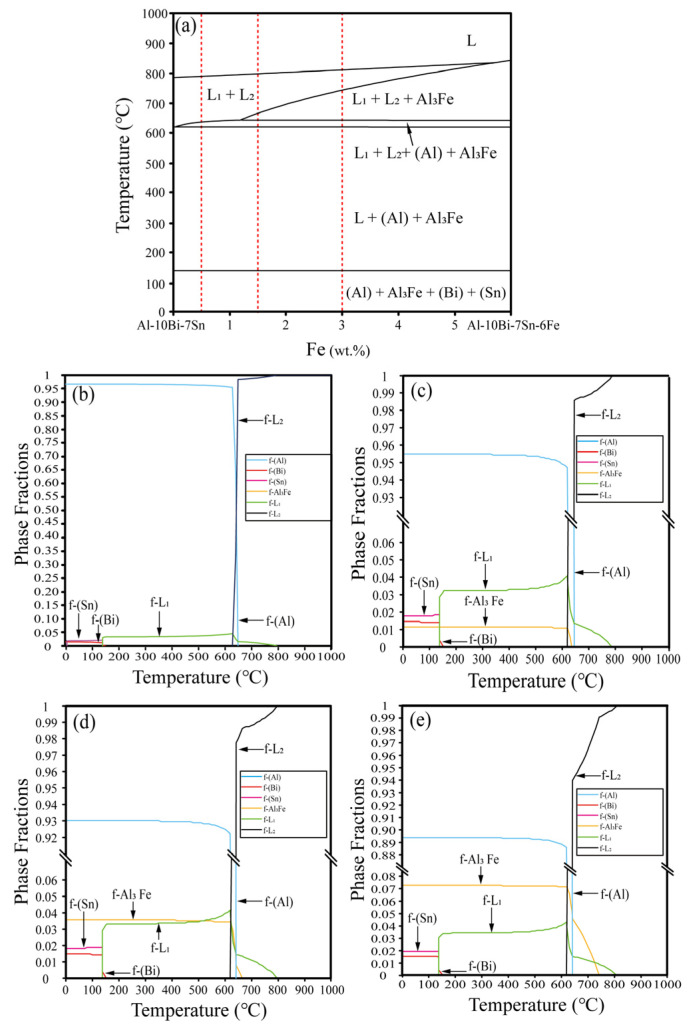
(**a**) Calculated vertical section diagram of Al-10Bi-7Sn-(0~6)Fe (wt.%) quasi-binary system and the phase fractions during solidification in (**b**) Al-10Bi-7Sn (wt.%) [33]; (**c**) Al-10Bi-7Sn-0.5Fe (wt.%); (**d**) Al-10Bi-7Sn-1.5Fe (wt.%); (**e**) Al-10Bi-7Sn-3Fe (wt.%).

The vertical section phase diagram of Al-10Bi-7Sn-(0~6)Fe (wt.%) was calculated by the phase diagram calculation method based on the thermodynamic database of the Al-based alloy established by the present research group, as presented in Figure 2, where liquid-phase L_1_ represented the liquid (Bi, Sn)-rich phase, and liquid-phase L_2_ represented the liquid Al-rich phase. In Figure 2a, the immiscible liquid-phase in this ternary system is obvious, where the liquid-phase region was characterized by the layer separation between L_1_ and L_2._ The previous results suggested that the compositions in liquid-phase miscibility gap regions formed the powders during the rapid cooling process of gas atomization. Therefore, four kinds of Al-based powders with the compositions of Al-10Bi-7Sn, Al-10Bi-7Sn-0.5Fe, Al-10Bi-7Sn-1.5Fe, and Al-10Bi-7Sn-3Fe (wt.%) were prepared, where the alloy compositions were designed to fall into the stable miscibility gap in the liquid state based on the calculated vertical section. The calculated mole fractions of each phase during solidification of the Al-10Bi-7Sn-xFe (x = 0, 0.5, 1.5, 3) wt.% system are presented in Figure 2b–e. It can be seen from these figures that the liquid-phase miscibility gap occurred during the cooling process of the alloy system. Further, in the two-phase region, the mole fraction of the (Bi, Sn)-rich phase was smaller than that of the Al-rich phase. However, the mole fraction of the (Bi, Sn)-rich phase increased as the temperature decreased, and the mole fraction of the Al-rich phase also decreased, which shows good agreement with the calculated phase diagram. The mole fractions of Al_3_Fe in the Al-10Bi-7Sn-xFe (x = 0.5, 1.5, 3) (wt.%) quaternary alloys were different, and the mole fractions of Al_3_Fe in descending order were as follows: Al-10Bi-7Sn-3Fe (wt.%), Al-10Bi-7Sn-1.5Fe (wt.%), and Al-10Bi-7Sn-0.5Fe (wt.%).

### 3.2. Morphology Observation

**Figure 3:**
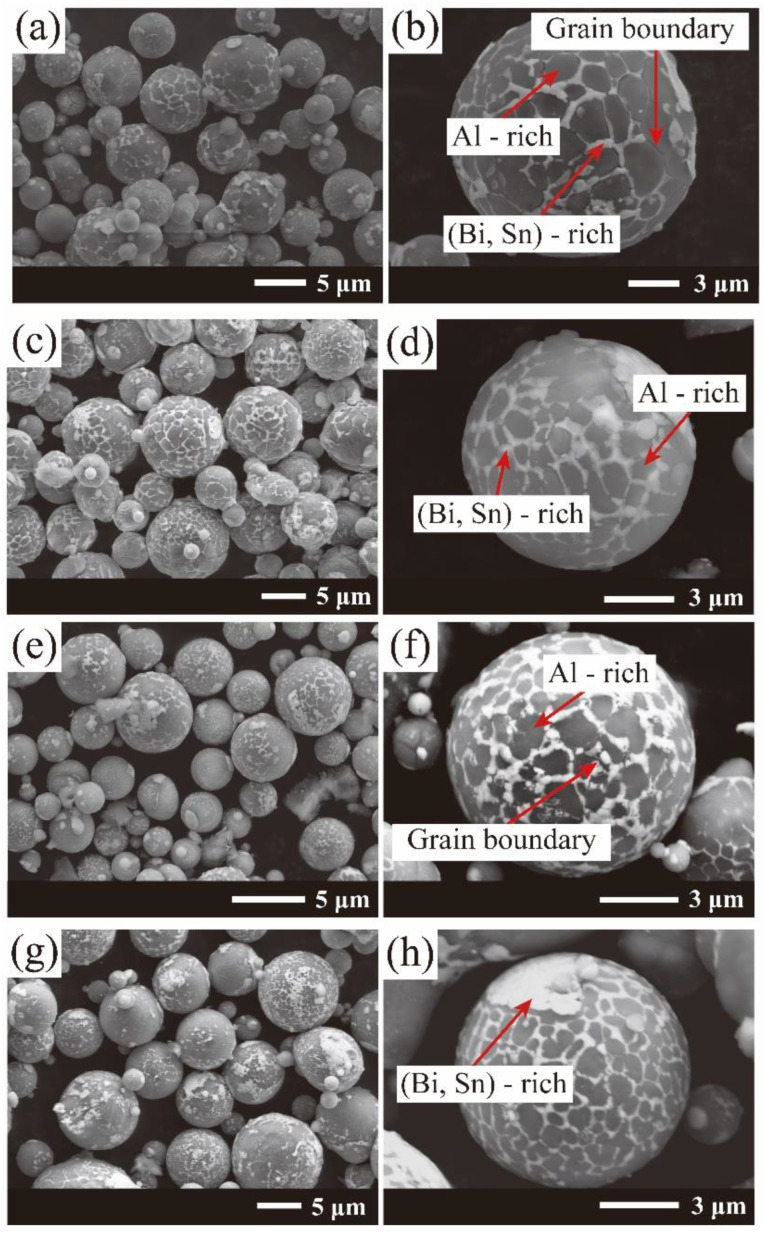
SEM images of the as-atomized Al-10Bi-7Sn-xFe (x = 0, 0.5, 1.5, 3) (wt.%) composite powders: (**a**,**b**) Al-10Bi-7Sn (wt.%) [33]; (**c**,**d**) Al-10Bi-7Sn-0.5Fe (wt.%); (**e**,**f**) Al-10Bi-7Sn-1.5Fe (wt.%); (**g**,**h**) Al-10Bi-7Sn-3Fe (wt.%).

The scanning electron microscopy (SEM) images with a low magnification of Al-based powders with different Fe contents are presented in Figure 3a,c,e,g. Most of the Al-10Bi-7Sn-xFe (x = 0, 0.5, 1.5, 3) (wt.%) composite powders were spherical with a discontinuous grid (Bi, Sn)-rich phase spreading throughout the grain boundaries of the Al-rich phase, which can be confirmed by EDX analysis. This is mainly because the L_1_(Al-rich) and L_2_(Bi/Sn-rich) liquid phases have large immiscible liquid-phases. When the L_2_(Bi/Sn-rich) phase has a low phase fraction, the L_2_(Bi/Sn-rich) phase cannot completely wrap the L_1_(Al-rich) core–shell structure. Thus, the microstructures of these (Bi, Sn)-rich phases are distributed dispersively in the Al-rich phase matrix [40]. Moreover, slight differences were found between the surface morphology of these powders. Typical SEM images with a high magnification of Al-10Bi-7Sn-xFe (x = 0, 0.5, 1.5, 3) (wt.%) powders are presented in Figure 3b,d,f,h. The surfaces of most of the Al-based composite powders were smooth and continuous, whereas cracks could be obviously detected on the surfaces of a few of the Al-10Bi-7Sn (wt.%) powders [33]. The formation of surface cracks in the composite powders was ascribed to the fact that the Al-based composite powders prepared by the gas atomization method were unstable and had high chemical reactivity [42]. Therefore, once the powders were exposed to air, they easily reacted with H_2_O in the air, thereby resulting in a rupture of the surface morphology.

**Figure 4:**
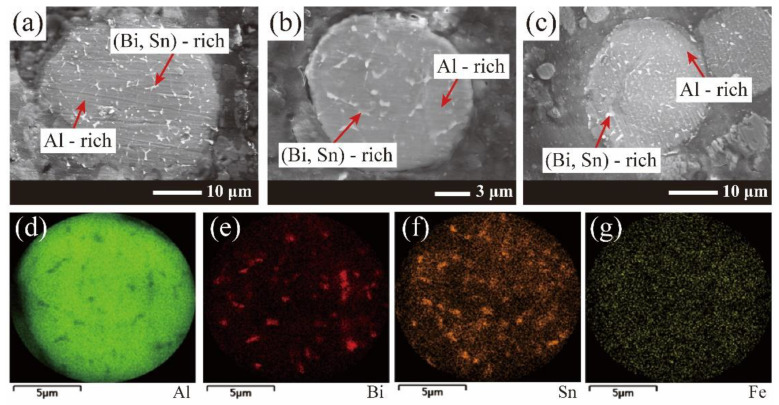
(**a**–**c**) SEM images of the cross-section morphology of Al-10Bi-7Sn-xFe (x = 0, 0.5, 1.5, 3) (wt.%) powders: (**a**) Al-10Bi-7Sn-0.5Fe (wt.%), (**b**) Al-10Bi-7Sn-1.5Fe (wt.%), (**c**) Al-10Bi-7Sn-3Fe (wt.%); (**d**–**g**) the EDS elemental mapping of Al-10Bi-7Sn-1.5Fe (wt.%) for (**d**) Al, (**e**) Bi, (**f**) Sn, (**g**) Fe.

The SEM image of the cross-section morphology and the EDS elemental mapping of the Al-10Bi-7Sn-xFe (x = 0, 0.5, 1.5, 3) (wt.%) composite powders’ internal structure are presented in Figure 4. According to the SEM image of the cross-sectional morphology, the Al-10Bi-7Sn-xFe (x = 0, 0.5, 1.5, 3) (wt.%) powders had a similar microstructure as the surface grid (Bi, Sn)-rich phase discontinuously distributed at the grain boundary of the Al-rich phase. Furthermore, the (Bi, Sn)-rich phase was also dispersed in the Al matrix in the form of tiny droplets. According to the EDS analysis of the elemental distribution, most Bi and Sn existed in the (Bi, Sn)-rich phase, and the trace-added Fe was evenly distributed throughout the cross-section. This demonstrated that all the elements were homogeneously distributed in the composites and would promote the formation of Al/metal micro-galvanic cells that could accelerate the hydrolysis reaction. The previous studies indicated that the core/shell microstructures of the Al-10Bi-7Sn-xFe (x = 0, 0.5, 1.5, 3) (wt.%) composite powders were attributed to both the differences in the volume fractions and the differences in the surface energies between the separated phases [43,44]. Under the extremely high cooling rate of gas atomization, there is a great temperature gradient in the molten droplets, where the influences of the differences in surface energies would play a greater role [45]. As the energy of the whole powder decreased, the (Bi, Sn)-rich phase with lower surface energies moved outward and occupied the powder surface. However, the volume of the (Bi, Sn)-rich phase was too small to wrap the whole surface of the composite powder. In addition, since the solidifying point of the composition of the (Bi, Sn)-rich phase was much lower than the Al-rich phase [35], with the temperature decreasing rapidly, the Al-rich phase first solidified, and the (Bi, Sn)-rich phase could only precipitate around the grain boundary of the outer layers of the powders.

**Figure 5:**
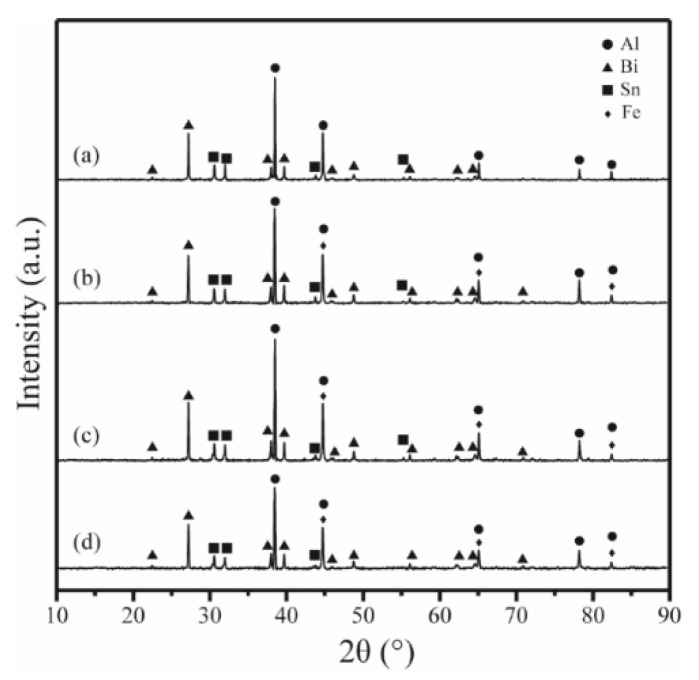
XRD patterns of the prepared composite powders: (**a**) Al-10Bi-7Sn (wt.%) [33]; (**b**) Al-10Bi-7Sn-0.5Fe (wt.%); (**c**) Al-10Bi-7Sn-1.5Fe (wt.%); (**d**) Al-10Bi-7Sn-3Fe (wt.%).

The X-ray diffraction patterns of the Al-10Bi-7Sn-xFe (x = 0, 0.5, 1.5, 3) (wt.%) composite powders are shown in Figure 5. As can be seen in the figure, the Al, Bi, and Sn diffraction peaks were obviously obtained in all the patterns. This was closely related to the fact that the Al, Bi, and Sn did not react with each other during gas atomization, which was determined by the limited solid solubility and lack of compound formed in the Al-Bi-Sn system [46]. The XRD result was in good agreement with previous research results [47]. Moreover, a comparison of the XRD patterns of the Al-10Bi-7Sn composite powder with those of the powders Al-10Bi-7Sn-0.5Fe (wt.%), Al-10Bi-7Sn-1.5Fe (wt.%), and Al-10Bi-7Sn-3Fe (wt.%) revealed that all Al diffraction peaks of the Al-Bi-Sn-Fe composite powders were slightly shifted to the left side after the addition of Fe. The result indicated that some Fe atoms may dissolve into the lattice of Al.

### 3.3. Hydrogen Generation Properties

**Figure 6:**
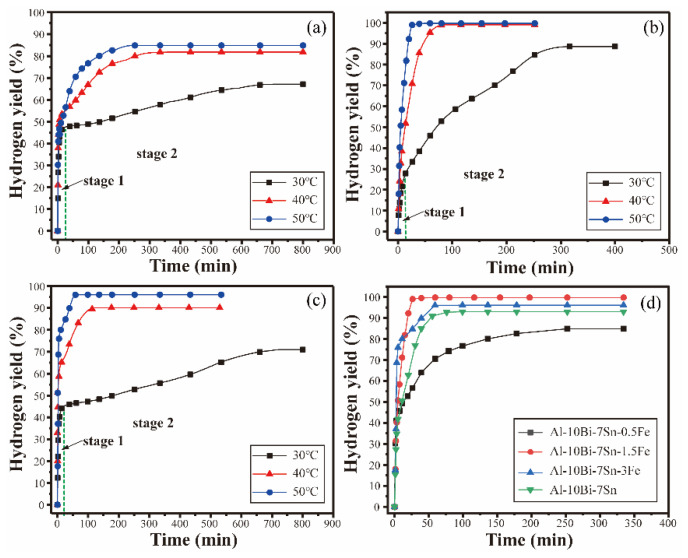
Hydrogen generation curves of the Al-Bi-Sn-Fe composite powders reacting with distilled water at 50 °C. (**a**) Al-10Bi-7Sn-0.5Fe (wt.%); (**b**) Al-10Bi-7Sn-1.5Fe (wt.%); (**c**) Al-10Bi-7Sn-3Fe (wt.%); (**d**) Al-10Bi-7Sn-xFe (x = 0, 0.5, 1.5, 3) (wt.%) composite powders [33].

The hydrogen generation properties of the composite powders while reacting with distilled water at different temperatures (30 °C, 40 °C, and 50 °C) were investigated, and the results are shown in Figure 6. As shown in Figure 6, the Al-10Bi-7Sn-xFe (x = 0, 0.5, 1.5, 3) (wt.%) composite powders exhibited superior hydrolysis characteristics, and the process could be divided into two stages. The initial stage (stage 1) was a fast-growing stage, and the conversion rate increased rapidly. Then, the next step was a dormant period (stage 2), which was a slowly growing phase with a long duration. As shown in Figure 6a, the initial stage of the reaction was a rapid hydrogen generation stage; the hydrogen yield increased rapidly to 47% within 50 min. Subsequently, the hydrolysis process entered stage 2; the hydrogen yield reached 66.8% at 650 min. Higher temperatures promoted the reactions; the reactions achieved higher conversion yields in smaller amounts of time. As the reaction temperature rose, there were still two reaction stages in the hydrolysis process of the Al-10Bi-7Sn-0.5Fe, Al-10Bi-7Sn-1.5Fe, and Al-10Bi-7Sn-3Fe (wt.%) composite powders at 40 °C and 50 °C. Notably, stage 2 was shortened, whereas stage 1increased correspondingly. The composite powders produced hydrogen rapidly at the instant of contact with water until the reaction stopped, which could complete all the reaction stages in a short time, and the final hydrogen yield was close to 100%.

The changes in the hydrogen generation curves of the sample Al-10Bi-7Sn-xFe (x = 0, 0.5, 1.5, 3) (wt.%) composite powders before and after the Fe addition were used to estimate the hydrogen generation properties. Figure 6d presents the hydrogen generation curves of the Al-10Bi-7Sn-xFe (x = 0, 0.5, 1.5, 3) (wt.%) powders in distilled water at 50 °C. Figure 6d reveals that no improvements were detected in the hydrogen yield of the Al-10Bi-7Sn-0.5Fe (wt.%) powders, whereas the hydrogen yield of the Al-10Bi-7Sn-1.5Fe (wt.%) and Al-10Bi-7Sn-3Fe (wt.%) powders was significantly higher than that of the Al-10Bi-7Sn (wt.%) composite powders. The Al-10Bi-7Sn-1.5Fe (wt.%) composite powders reacting immediately with distilled water showed the best hydrogen production properties. This phenomenon could be expounded by combining the calculated phase fractures of the Al-10Bi-7Sn-xFe (x = 0, 0.5, 1.5, 3) (wt.%) alloy powder preparation.

The hydrogen generation properties of the Al-10Bi-7Sn-0.5Fe (wt.%) composite powder, compared with the Al-10Bi-7Sn (wt.%) composite powder, did not show a significant improvement. This was mainly due to the precipitation amount of Al_3_Fe in Al-10Bi-7Sn-0.5Fe (wt.%) powder that was far lower than the calculated value, and the electrochemical effect had little effect in promoting hydrogen production. Meanwhile, the Fe addition strengthened the Al matrix phase through Al(Fe) solution strengthening, and the hydrogen-production reaction of aluminum water stopped before the complete reaction of Al, which is always obvious at low temperatures. However, with the increase in iron content, the precipitation amount of the Al_3_Fe phase also increases, and Al_3_Fe precipitation becomes the preferred site for cathode reaction, resulting in local pH rise of nearby solution [46]. Local alkalization accelerates the evolution of corrosive hydrogen around Al_3_Fe by reaction Equations (3)–(5), leading to the excellent hydrogen-production performance of the Al-10Bi-7Sn-1.5Fe (wt.%) and Al-10Bi-7Sn-3Fe (wt.%) powders. On the other hand, with the excessive addition of iron, a large amount of Al_3_Fe will be precipitated in the alloy, and the Al matrix will be improved by strengthening the second phase [47]. This enhancement results in no cracks in the hydrolysis process of the Al-10Bi-7Sn-xFe (x = 0, 0.5, 1.5, 3) (wt.%) composite powder, which reduces the contact area between Al and water and hinders the hydrolysis reaction of the powder. Therefore, the addition of Fe has a complex effect on the hydrogen properties of Al-Bi-Sn composite powders, and only a certain amount of Fe can obtain the optional properties.

**Figure 7:**
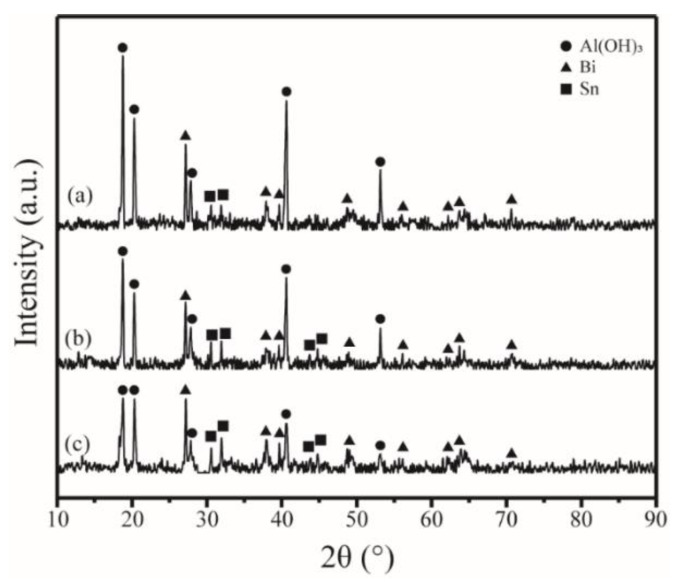
XRD patterns of composite powders after hydrogen generation: (**a**) Al-10Bi-7Sn-0.5Fe (wt.%); (**b**) Al-10Bi-7Sn-1.5Fe (wt.%); (**c**) Al-10Bi-7Sn-3Fe (wt.%).

Figure 7 exhibits the XRD pattern of the Al-10Bi-7Sn-xFe (x = 0.5, 1.5, 3) (wt.%) composite powders after hydrogen generation. Compared with the XRD pattern of the Al-10Bi-7Sn-xFe (x = 0.5, 1.5, 3) wt.% composite powders before hydrogen generation (Figure 5), on the one hand, the diffraction peaks of Al disappeared, whereas the diffraction peaks of Al(OH)_3_ became stronger, because, during the hydrolysis of Al, Al(OH)_3_ in addition to H_2_ was generated. On the other hand, there was no obvious change in the diffraction peaks of Bi and Sn, because, during the hydrolysis process, Bi and Sn hardly participate in the reaction of hydrogen production by hydrolysis. The above results were consistent with the previous research results by Liu et al. [35]. Particularly, in Figure 7, we can see that the Fe diffraction peaks disappear; this may be because the product of Al participating in the hydrolysis process is Al(OH)_3_, which is accompanied by a weight change, and the iron content is therefore so low that it is difficult to detect. In addition, in the hydrolysis process, along with the fragmentation and dispersion of the composite powder structure, the detectability of Fe diffraction peaks is further reduced.

**Figure 8:**
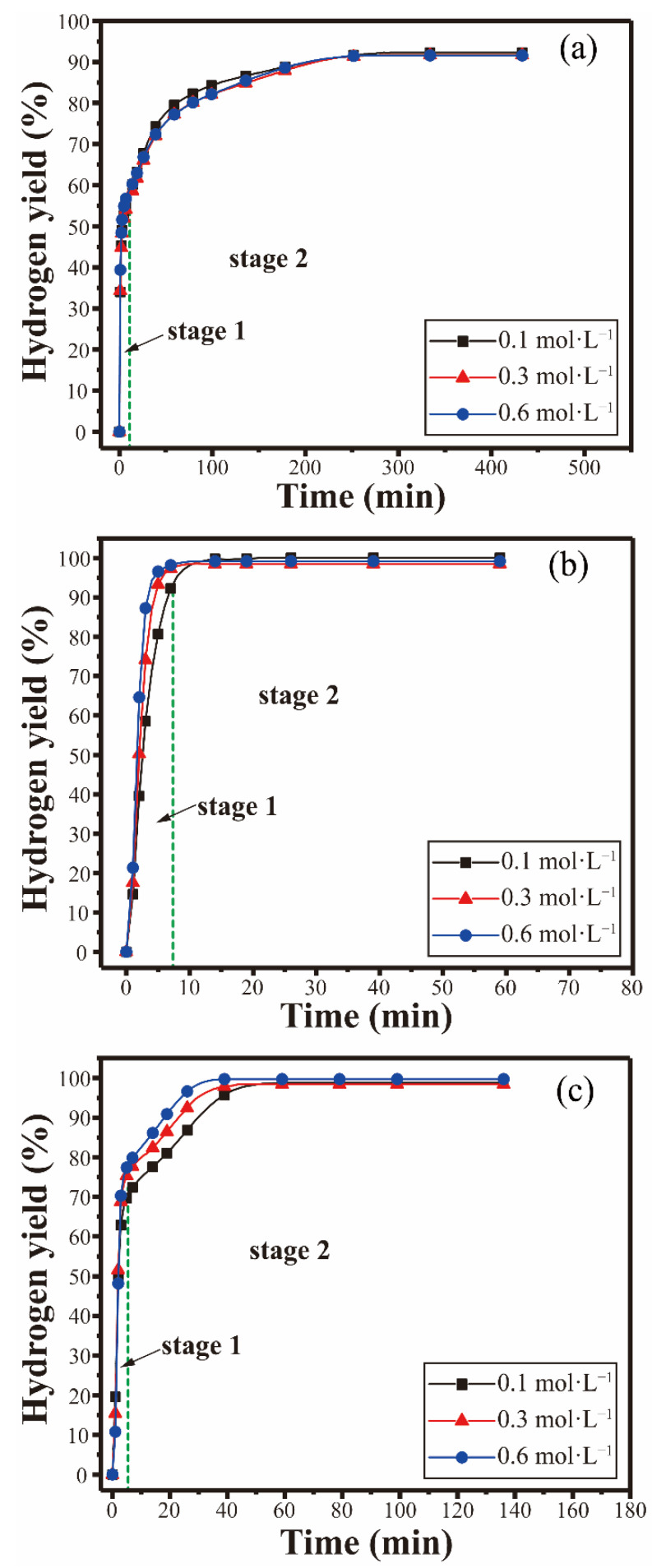
Hydrogen generation curves of the Al-Bi-Sn-Fe composite powders reacting with NaCl solutions of different concentrations at 50 °C: (**a**) Al-10Bi-7Sn-0.5Fe (wt.%); (**b**) Al-10Bi-7Sn-1.5Fe (wt.%); (**c**) Al-10Bi-7Sn-3Fe (wt.%).

The hydrogen generation properties in different reaction media were investigated. The hydrogen generation properties of the Al-10Bi-7Sn-xFe (x = 0.5, 1.5, 3) (wt.%) composite powders reacting with NaCl in different concentrations at 50 °C were investigated, and the results are shown in Figure 8. The hydrogen-production properties of the Al-10Bi-7Sn-xFe (x = 0.5, 1.5, 3) (wt.%) powders in NaCl solution were significantly improved. In the 0.1 mol·L^−1^ NaCl solution, the Al-10Bi-7Sn-xFe (x = 0.5, 1.5, 3) (wt.%) powders generated hydrogen rapidly after contact, the final hydrogen yield of Al-10Bi-7Sn-0.5Fe (wt.%) reached 92% in 250 min, Al-10Bi-7Sn-1.5Fe (wt.%) reached 99% in 15 min, and Al-10Bi-7Sn-0.5Fe (wt.%) reached 96% in 40 min. With the NaCl concentration increasing to 0.3 mol·L^−1^ and 0.6 mol·L^−1^, the Al-10Bi-7Sn-xFe (x = 0.5, 1.5, 3) (wt.%) powders still have a high hydrogen generation rate and final hydrogen yield.

**Figure 9:**
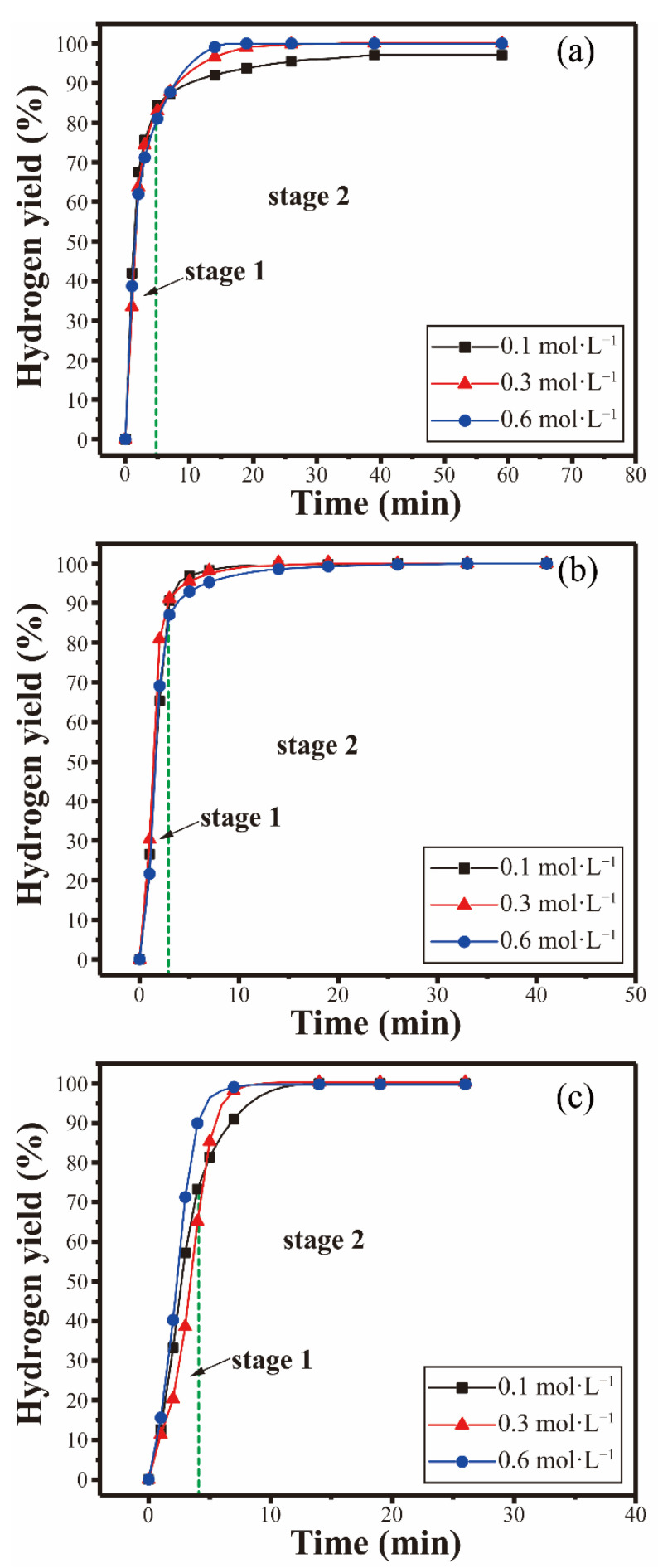
Hydrogen generation curves of the Al-Bi-Sn-Fe composite powders reacting with CaCl_2_ solutions of different concentrations at 50 °C: (**a**) Al-10Bi-7Sn-0.5Fe (wt.%); (**b**) Al-10Bi-7Sn-1.5Fe (wt.%); (**c**) Al-10Bi-7Sn-3Fe (wt.%).

The hydrogen generation properties of the Al-10Bi-7Sn-xFe (x = 0.5, 1.5, 3) (wt.%) composite powders reacting with CaCl_2_ in different concentrations at 50 °C were investigated, and the results are shown in Figure 9. Al-10Bi-7Sn-0.5Fe, Al-10Bi-7Sn-1.5Fe, and Al-10Bi-7Sn-3Fe (wt.%) could all produce hydrogen at the moment of contact with 0.1 mol·L^−1^ CaCl_2_ solution, and the hydrogen production was rapid until the powder was completely reversed. All could generate hydrogen rapidly in 0.1 mol·L^−1^ CaCl_2_ solution, with the final hydrogen yield nearly 100%. As concentration increased to 0.3 mol·L^−1^ and 0.6 mol·L^−1^, Al-10Bi-7Sn-xFe (x = 0.5, 1.5, 3) (wt.%) composite powders maintained excellent hydrogen production properties. The hydrolysis reaction could complete in 15 min, and the hydrogen is close to 100%.

As can be seen from the experimental results, the hydrogen production performance greatly improved when the Al-10Bi-7Sn-xFe (x = 0.5, 1.5, 3) (wt.%) composite powders reacted with NaCl and CaCl_2_ solutions. This is mainly due to the fact that in NaCl and CaCl_2_ solutions, the corrosion potentials of Fe elements and Al_3_Fe compounds in the composite powder are higher than those of the Al-rich phase in NaCl and CaCl_2_ solutions, and the increasing difference in corrosion potentials will lead to a stronger effect of the miniature galvanic cells [48]. In addition, the existence of Cl^−^ increases the conductivity of the solution, and accelerates the corrosion and hydrogen evolution, thus increasing the hydrolysis reaction rate and hydrogen conversion rate [49].

### 3.4. Anti-Oxidant Properties

**Figure 10:**
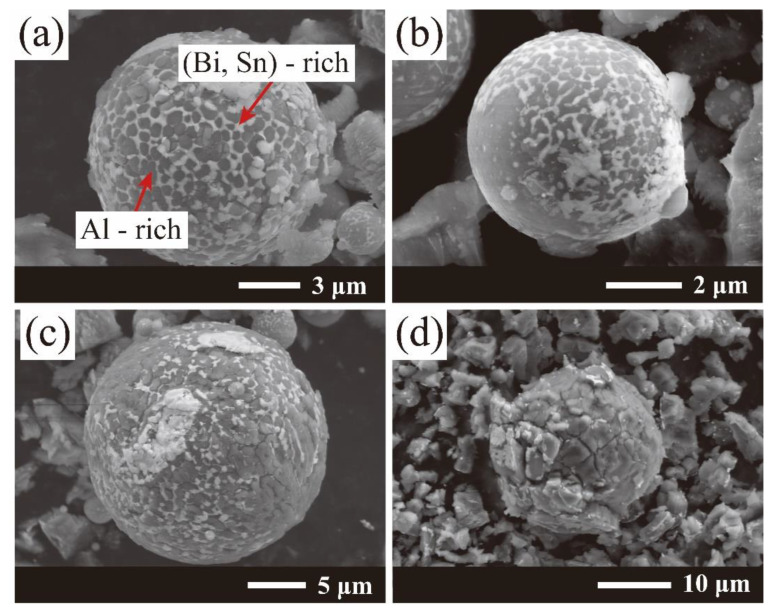
Surface morphologies of the Al-10Bi-7Sn-xFe (x = 0, 1.5) (wt.%) composite powders exposed to air for different times (25 °C and 60 RH%): (**a**) Al-10Bi-7Sn-1.5Fe (wt.%), 12 h; (**b**) Al-10Bi-7Sn-1.5Fe (wt.%), 24 h; (**c**) Al-10Bi-7Sn-1.5Fe (wt.%), 72 h; (**d**) Al-10Bi-7Sn (wt.%), 12 h [33].

Traditionally, Al-based hydrogen generation materials are easy to passivate in air-exposed conditions, especially since the humidity of the air could accelerate the oxidation and inactivation processes. The anti-oxidation property has a further vital performance in metal hydrolysis reaction applications. Thus, it is vital to monitor the activity of the powders when exposed to the air. The anti-oxidation performance of the prepared Al-10Bi-7Sn (wt.%) and Al-10Bi-7Sn-1.5Fe (wt.%) composite powders was evaluated by exposing them to the air at a constant temperature and humidity chamber (25 °C and 60 RH%) for different times. Figure 10 gives the SEM image of the surface morphologies of the Al-10Bi-7Sn-1.5Fe (wt.%) powders after being placed for 12, 24, and 72 h, and the Al-10Bi-7Sn powders after being placed for 24 h. No obvious changes were found in the excellent spherical degree of the Al-10Bi-7Sn-1.5Fe (wt.%) composite powders. After a period of continuous exposure to the air, only a small amount of the (Bi, Sn)-rich phase was shed, revealing the grain boundary of the Al-rich phase and forming a fine crack along the grain boundary, as shown in Figure 10a–c. Meanwhile, when the Al-10Bi-7Sn composite powder was exposed to the air for 12 h, the expansion of the composite powder along the grain boundary and the byproduct fell off from the surface. This was closely related to moisture intruding into the crack on the surface of the Al-10Bi-7Sn powders, leading to a local hydrolysis reaction.

**Figure 11:**
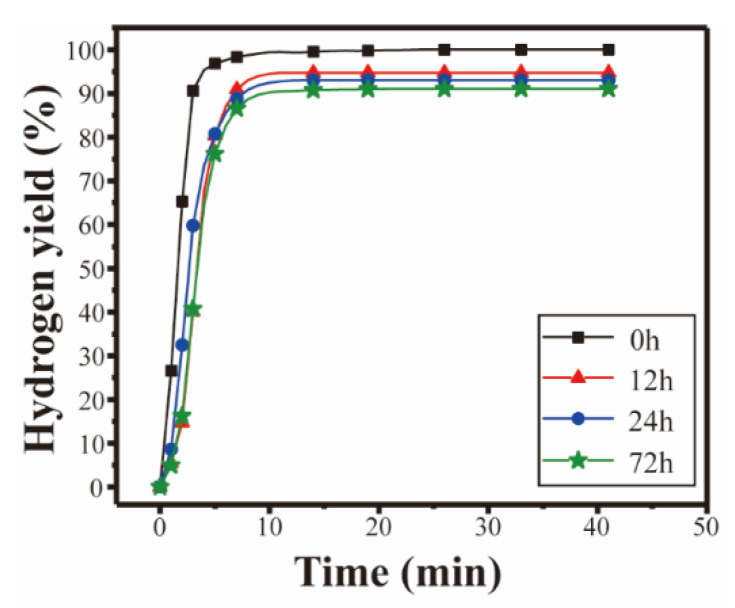
Hydrogen generation curves of the Al-10Bi-7Sn-1.5Fe (wt.%) composite powders exposed to air for different times reacting with 0.1 mol·L^−1^ CaCl_2_ solution at 50 °C.

The hydrogen generation properties of the composite powders after storing for different hours while reacting with CaCl_2_ solution were investigated, and the results are shown in Figure 11. As shown in Figure 11, after 12 h of exposure, the hydrogen yield of the Al-10Bi-7Sn-1.5Fe (wt.%) powders was 94.7% when they reacted with 0.1 mol·L^−1^ CaCl_2_ solution, which was 5% lower than that of the powders not exposed to air. With exposure time prolonged to 24 and 72 h, the hydrogen yield decreased to 93% and 91%. These results suggested that the hydrogen generation properties of the Al-10Bi-7Sn-1.5Fe (wt.%) composite powders included excellent anti-oxidation properties. This is mainly because the surface, with less small cracking in the Al-10Bi-7Sn-1.5Fe (wt.%) powders, effectively isolates the air moisture and prevents the water vapor from penetrating into the powder. Furthermore, when the Al-10Bi-7Sn-1.5Fe (wt.%) powders reacted with CaCl_2_ solution, the Cl^-^ in the solution could easily penetrate the oxide layer on the surface of the powders [50], then making contact with the fresh internal Al, building the Al-Al_3_Fe micro galvanic cell, thus promoting the rapid hydrolysis of the Al-10Bi-7Sn-1.5Fe (wt.%) powders to generate hydrogen. The addition of Fe to aluminum-based hydrogen production materials will become very promising in terms of the activity retention of Al-Bi-Sn composite powders exposed to air.

### 3.5. Mechanism Investigation

**Figure 12:**
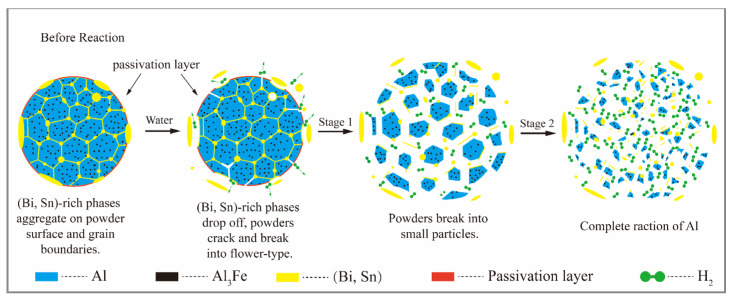
Schematic illustration of the reaction mechanisms of the Al-10Bi-7Sn-xFe (x = 0.5, 1.5, 3) (wt.%) composite powders reacting with distilled water at 30 °C.

According to previous studies, the Al-Bi-Sn composite powder has a reaction process of separating from the grain boundary and forming a flower-like structure [34]. The fresh Al surface is exposed to water, resulting in a severe hydrolysis reaction and high hydrogen production efficiency. A schematic illustration of the reaction mechanisms of the Al-10Bi-7Sn-xFe (x = 0.5, 1.5, 3) (wt.%) composite powders reacting with distilled water at 30 °C is presented in Figure 12. Like the flower-blooming reaction described above for the Al-Bi-Sn powders, the Fe addition also promotes hydrogen production reactions. This is mainly due to Al_3_Fe compounds formed in the equilibrium solidification structure of the Al-10Bi-7Sn-xFe (x = 0.5, 1.5, 3) (wt.%) alloys and their corrosion potential (-1.12 VSCE), which was higher than that of Al (-1.79 VSCE). The galvanic effect could be formed between the Al_3_Fe and Al matrix in the powders, thus accelerating the hydrolysis of Al to generate hydrogen [41,51]. The electrochemical corrosion of the Al_3_Fe and Al matrix can be expressed as follows:

O_2_ + H_2_O + 4e^−^ → 4OH^−^ (3)

2Al − 3e^−^ + 4OH^−^ → Al (OH)_3_ (4)

2H_2_O + 2e^−^ → H_2_ + 2OH^−^ (5)

Actually, Al_3_Fe diffraction peaks could not be obtained in the XRD pattern (Figure 5) due to the low precipitation of Al_3_Fe. The content of Al_3_Fe is very low—less than 7%, according to the phase diagram, as shown in Figure 2. According to EDS mapping, the Fe is diffusely distributed, as can be seen in Figure 4g. In addition, it seemed that the cooling rate in the process of pulverization by atomization inhibited the precipitation of the Al_3_Fe phase to some extent [33].

Further, in the Al-Fe alloy, the Al matrix can be strengthened by Al(Fe) solution strengthening, which may improve the oxidation resistance of the Al-Bi-Sn alloy powder in the air by strengthening the strength of the shell–core structure of the alloy powder by gas atomization.

## 4. Conclusions

In this work, activated Al-based hydrogen production powders with Bi, Sn, and Fe additives were prepared by ultrasonic gas atomization method. The Fe-additive enhanced the immiscible liquid-phase between the Al-rich phase and the (Bi, Sn)-rich phase. The Fe-additive also dissolved in the liquid aluminum-rich phase. During the solidification process, it mainly formed an Al(Fe) solid solution, as well as partially forming an Al_3_Fe precipitated phase. On this basis, the hydrogen generation properties of the composite powders in different aqueous media were studied. The results suggested that the Al-10Bi-7Sn-xFe (x = 0, 0.5, 1.5, 3) (wt.%) composite powder showed excellent hydrogen production properties, and Al-10Bi-7Sn-1.5Fe powders had the best performance. Notably, the hydrogen production properties of Al-10Bi-7Sn-xFe (x = 0.5, 1.5, 3) (wt.%) composite powders greatly improved in NaCl solutions and CaCl_2_ solutions. Furthermore, the Al-10Bi-7Sn-1.5Fe (wt.%) powders also showed an excellent anti-oxidant property. Even when exposed to air for 72 h, the Al-10Bi-7Sn-1.5Fe (wt.%) powders can still hydrolyze rapidly, leading to a hydrogen yield of 91% in 10 min.

The experimental results showed that the addition of iron additives in Al-Bi-Sn composite powder could give the composite powder the advantages of high reactivity, low price, and strong oxidation resistance, which would be widely valued in the direction of mobile hydrogen sources for rapid hydrogen production.

## Data Availability

Not applicable.
